# p53 represses human papillomavirus type 16 DNA replication via the viral E2 protein

**DOI:** 10.1186/1743-422X-5-5

**Published:** 2008-01-11

**Authors:** Craig Brown, Anna M Kowalczyk, Ewan R Taylor, Iain M Morgan, Kevin Gaston

**Affiliations:** 1 Department of Biochemistry, School of Medical School, University of Bristol, Bristol, UK; 2 Institute of Comparative Medicine, University of Glasgow, Glasgow, UK

## Abstract

**Background:**

Human papillomavirus (HPV) DNA replication can be inhibited by the cellular tumour suppressor protein p53. However, the mechanism through which p53 inhibits viral replication and the role that this might play in the HPV life cycle are not known. The papillomavirus E2 protein is required for efficient HPV DNA replication and also regulates viral gene expression. E2 represses transcription of the HPV E6 and E7 oncogenes and can thereby modulate indirectly host cell proliferation and survival. In addition, the E2 protein from HPV 16 has been shown to bind p53 and to be capable of inducing apoptosis independently of E6 and E7.

**Results:**

Here we use a panel of E2 mutants to confirm that mutations which block the induction of apoptosis via this E6/E7-independent pathway, have little or no effect on the induction of apoptosis by the E6/E7-dependent pathway. Although these mutations in E2 do not affect the ability of the protein to mediate HPV DNA replication, they do abrogate the repressive effects of p53 on the transcriptional activity of E2 and prevent the inhibition of E2-dependent HPV DNA replication by p53.

**Conclusion:**

These data suggest that p53 down-regulates HPV 16 DNA replication via the E2 protein.

## Background

Human papillomaviruses (HPVs) are non-enveloped, small double-stranded DNA tumour viruses that are strictly epitheliotropic, infecting cutaneous or mucosal epithelial cells typically of the anogenital tract, hands or feet [1,2]. To date over 100 types of HPV have been identified and these viruses cause a range of diseases from benign hyperproliferative warts to epithelial tumours. Many HPV types infect the genital tract and these viruses can be separated into two groups based on their oncogenic potential: high-risk HPV and low-risk HPV [1,2]. HPV types in the high-risk group are associated with the development of cancers of the anogenital tract, whereas low-risk HPVs are associated with benign genital warts. DNA from high-risk HPV types (predominantly types 16 and 18) is found in more than 99% of cervical squamous cell cancer cases worldwide [3]. High-risk HPVs have also been linked to other cancers including vulvar and penile cancers and cancer of the oropharynx [4,5].

The HPV genome contains 8 open reading frames (ORFs) that encode the non-structural proteins required for viral replication and the structural proteins that form the viral coat. Expression of these ORFs is controlled by a non-coding Long Control Region (LCR) that contains a complex array of transcription factor binding sites and the viral origin of replication [6]. The E6 and E7 ORFs encode oncoproteins that impact upon multiple regulatory pathways in the host cell in order to facilitate completion of the viral life cycle. Co-expression of the HPV E6 and E7 proteins from high-risk HPV types can efficiently immortalise primary human keratinocytes [7]. The E6 and E7 from these viruses interact with the tumour suppressor proteins p53 and pRb, respectively, as well as with many other cellular proteins [8]. E7 proteins from high-risk HPV types bind to pRB and increase cell proliferation by disrupting pRB-E2F complexes and by targeting pRB for degradation by the proteasome [9-11]. HPV E6 proteins from high-risk HPV types bind to p53 and target this protein for degradation by the proteasome [12,13]. This significantly reduces the steady-state level of p53 within the infected cell leading to the abrogation of the p53-mediated apoptosis that would otherwise accompany the expression of E7. The E6 and E7 proteins from low-risk HPV types bind to these and other cellular targets with reduced affinity or at least with a different outcome in terms of oncogenesis [9,14].

The HPV E2 protein plays an important role in viral replication and in the regulation of HPV gene expression [15]. E2 binds to four sites within the HPV LCR and via a protein-protein interaction, increases binding of the viral E1 protein to the origin of replication [16,17]. E1 is an ATP-dependent helicase which unwinds the double-stranded viral DNA and recruits cellular factors that allow replication to proceed [18,19]. In HPV-infected cells, the binding of E2 to the LCR is thought to repress HPV gene expression. In this way E2 contributes to the control of cell proliferation by regulating the expression of E6 and E7. However, in cervical carcinomas the HPV genome often becomes integrated into the host genome resulting in loss of E2 expression [20]. This leads to increased levels of E6 and E7 and, as a consequence, increased cell proliferation and presumably increased tumourigenesis. When E2 is re-introduced into these HPV-transformed cells experimentally, the control of cell proliferation is reintroduced resulting in reduced cell proliferation, increased cell senescence and increased apoptosis [21-23]. In addition, recent work from a number of laboratories has shown that the E2 proteins from high-risk HPV types can also induce apoptosis independently of E6 and E7 [24,25]. For instance, the HPV 16 E2 protein has been shown to induce apoptosis in a number of HPV negative cell lines [24,26]. Furthermore, the HPV 16 E2 protein can interact directly with p53 [27] and recent studies have shown that mutations in the HPV 16 E2 protein that prevent the DNA binding domain of E2 from interacting with p53, block E2-induced apoptosis in HPV-negative cells that express wild type p53 [26]. Over-expression of p53 has been shown to repress HPV 11 DNA replication although the mechanism underlying this phenomenon has yet to be determined [28,29]. Here we show that the interaction of p53 with E2 inhibits HPV 16 DNA replication as well as modulating the transcriptional activity of the HPV 16 E2 protein.

## Results

### Mutagenesis of the HPV 16 E2 protein

**Figure 1:**
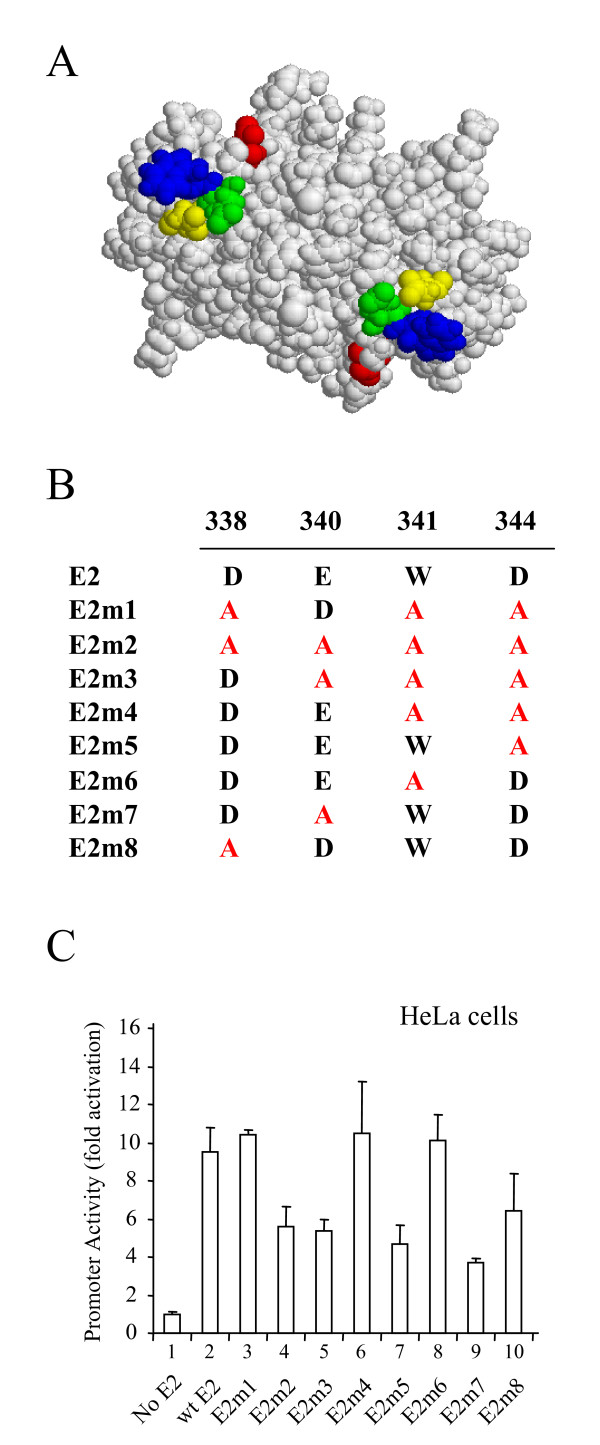
**Mutagenesis of the HPV 16 E2 protein**. (A) A molecular model of the dimeric DNA binding domain of the HPV 16 E2 protein [49] produced using RasMol 2.7.3.1 [50] and showing the amino acids mutated in this study: D338 (red), E340 (green), W341 (blue) and D344 (yellow). (B) The table shows the amino acids changes made in mutants E2m1 to E2m8. E2m1 was formerly referred to as E2p53m. (C) The graph shows the levels of luciferase activity found in HeLa cell extracts 24 hrs after transient co-transfection with an E2-responsive reporter plasmid and plasmids expressing the E2 proteins described above. Promoter activity was normalized with respect to transfection efficiency using a co-transfected plasmid expressing Renilla luciferase and is shown as fold activation over the reporter alone. The results are the average and standard deviation of four experiments.

The C-terminal DNA-binding domain of the HPV 16 E2 protein is important for binding to p53; residues 339–351 are essential for the interaction while residues 307–339 play a contributory role [27]. In an attempt to identify the specific amino acids in E2 that are involved in the interaction with p53, a molecular model of the complex was constructed [26]. The structure of the C-terminal domain of HPV 16 E2 has been determined by X-ray crystallography [30]. The crystal structure of the complex formed between p53 and 53BP2 [31] was used as a guide to build a molecular model of the E2-p53 complex. Model building identified four amino acids in E2 (D338, E340, W341 and D344) that can be superimposed on amino acids in 53BP2 important for binding to p53 (See Fig. 1A). Three of these residues (D338, W341 and D344) were previously mutated to alanine to create the mutant E2p53m [26], hereafter referred to as E2m1. This mutant of E2 is deficient in binding to p53 and fails to induce apoptosis in HPV-negative cells expressing wild type p53 [26]. To further investigate which residues in E2 are important in binding to p53, we have created a series of site-directed mutants in which D338, E340, W341 and D344 have been mutated to alanine either alone or in combinations (Fig. 1B).

To compare the abilities of the wild type and mutated E2 proteins to activate transcription, plasmids expressing each protein were transiently co-transfected into HeLa cells with an E2-responsive reporter construct consisting of six E2 binding sites upstream of the minimal thymidine kinase promoter and firefly luciferase gene [32]. Although HeLa cells are HPV-transformed, the viral genome is integrated into the host genome and expression of the E2 protein is lost [33]. The results of the experiments are shown in Figure 1C. In the absence of an E2 expression vector there is very little reporter activity (Fig. 1C, column 1). In the presence of the co-transfected wild type E2 expression vector, promoter activity is increased around 10 fold (Fig. 1C, column 2). As can be seen from the data, all of the mutated E2 proteins activate transcription in these cells. This confirms that all of the p53 interaction mutants are expressed and that none of the mutations dramatically affect the stability of the protein. However, mutants m2, m3, m5, and m7 activate transcription to a lesser extent than the other mutants and wild type E2.

### E2-induced apoptosis

**Figure 2:**
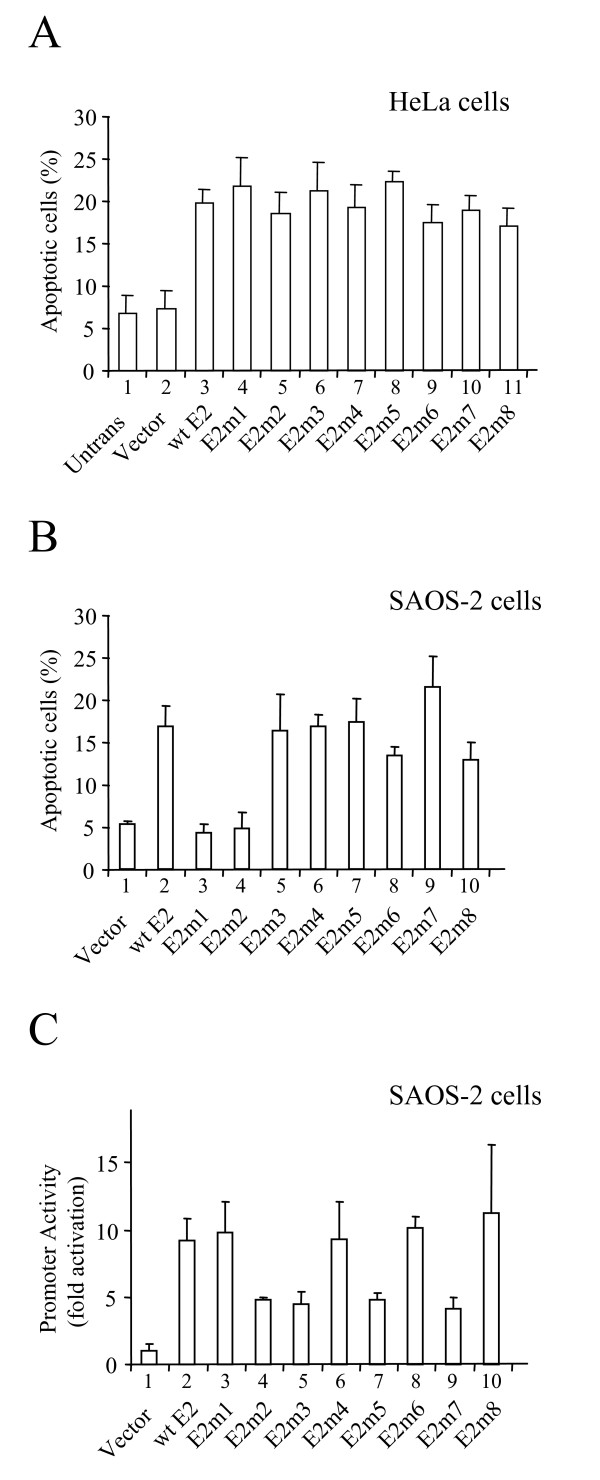
**The induction of apoptosis in HPV-transformed and non-HPV-transformed cells**. (A) HPV-transformed HeLa cells growing on coverslips were transiently co-transfected with plasmids expressing the E2 proteins shown in the figure and a plasmid expressing GFP. After 30 hours the cells were fixed and stained and the number of apoptotic cells in the transfected (green) population determined by counting. The data represent the mean and standard deviation of four independent experiments. (B) The experiment described above was repeated in non-HPV-transformed SAOS-2 cells. In this case 200 ng of the p53 expressing plasmid pCB6-p53 was included in each co-transfection. The data shown is the mean and standard deviation of four independent experiments. (C) The graph shows the luciferase activity found in SAOS-2 cell extracts 24 hrs after transient co-transfection with an E2-responsive reporter plasmid and plasmids expressing the E2 proteins described in Figure 1B. Promoter activity was normalized with respect to transfection efficiency and is shown as fold activation over the reporter alone. The results are the average and standard deviation of four experiments.

The HPV 16 E2 protein can induce apoptosis in HPV-transformed cells via the regulation of E6/E7 expression and via a direct interaction with p53. However, in non-HPV-transformed cells E2 is only able to induce apoptosis via the second pathway. To investigate the ability of each E2 mutant described above to induce apoptosis we first expressed the proteins in HPV-transformed cells. HeLa cells growing on coverslips were transiently co-transfected with plasmids that express either the wild type E2 protein or one of the E2 mutants and a plasmid expressing green fluorescent protein (GFP). GFP expression allows transfected cells to be identified and these cells were assessed for two characteristic features of apoptotic cells, chromatin condensation and membrane blebbing, using bisbenzimide staining and GFP flourescence, respectively. The percentage of cells undergoing apoptosis was then determined by counting at least 100 transfected cells from several locations on each coverslip and recording how many of the cells exhibit apoptotic morphology. We have shown previously that this is a robust method for the analysis of E2-induced apoptosis [34]. Untransfected HeLa cells and HeLa cells transfected with the empty vector show a background level of apoptosis of around 7% (Fig. 2A, columns 1 and 2). When wild type HPV 16 E2 is expressed in these cells the level of apoptosis rises to around 20% of the transfected population (Fig. 2A, column 3). All of the mutant E2 proteins examined in this experiment induce apoptosis in HeLa cells to around this level (Fig. 2A, columns 4–11). Since all of the mutants induce apoptosis to around the same level, these data suggest that the proteins are expressed at equivalent levels.

To investigate the ability of each of the E2 mutants to induce apoptosis in the absence of E6 and E7, we repeated the experiments described above in HPV-negative SAOS-2 cells. SAOS-2 cells are p53-null and we have shown previously that under the conditions used in these experiments, the HPV 16 E2 protein does not induce apoptosis in these cells unless it is co-expressed with p53 [24]. Wild type E2 and each of the mutants described above were transiently co-transfected into SAOS-2 cells with plasmids expressing p53 and GFP and the number of apoptotic cells determined exactly as described above. Cells co-transfected with the empty E2 vector and a low amount of the p53 expressing plasmid show a background level of apoptosis of around 6% (Fig. 2B, column 1). Co-expression of wild type HPV 16 E2 and p53 in these cells results in an increase in the level of apoptotic cells to around 18% of the transfected population (Fig. 2B, column 2). Interestingly, E2m1 and E2m2 fail to induce apoptosis in these cells (columns 3 and 4). In contrast, the remaining mutants induce apoptosis to levels comparable to that induced by wild type E2 (columns 5–10). As shown above, the E2m1 and E2m2 mutants are both capable of inducing apoptosis (Fig. 2A) and activating transcription (Fig. 1C) in HeLa cells. These results suggest that these two mutants are unable to directly activate p53 to induce apoptosis in this HPV-negative cell line. However, a trivial explanation for these results could be that these two mutants are expressed in HeLa cells but not in SAOS-2 cells. In order to rule out this possibility we examined the ability of each mutant to activate transcription in SAOS-2 cells. Transient transfection of SAOS-2 cells with the E2-responsive reporter described above results in very little reporter activity (Fig. 2C, column 1). However, as expected wild type E2 activates reporter activity around 10 fold in these cells (Fig. 2C, column 2). The E2 mutants all activate transcription in these cells confirming that they are all expressed (Fig. 2C, columns 3–10). However, as seen in HeLa cells, mutants m2, m3, m5, and m7 activate transcription to a lesser extent than the other mutants and wild type E2. This again indicates that the differences in transcription activation seen in HeLa and SAOS-2 cells are not due to differences in protein expression levels. However, we were unable to detect any of these proteins using E2-specific antibodies (not shown) and we are therefore unable to confirm this conclusion.

### Modulation of the transcriptional activity of E2 by p53

**Figure 3:**
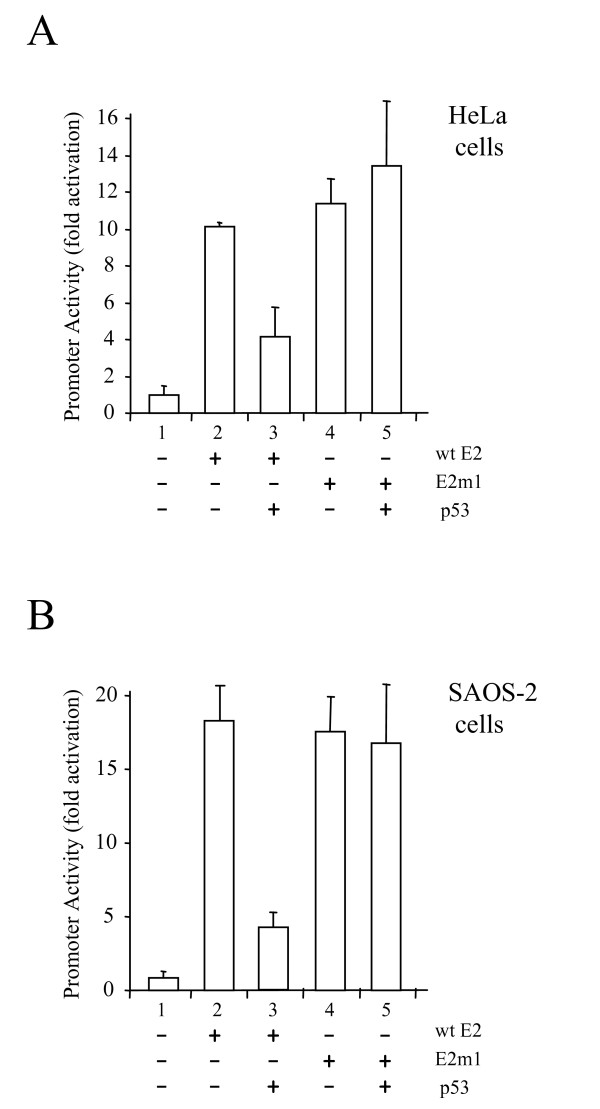
**p53 represses E2-induced transcription**. The graphs show the levels of luciferase activity found in (A) HeLa and (B) SAOS-2 cell extracts 24 hrs after transient co-transfection with an E2-responsive reporter plasmid and plasmids expressing E2 or E2m1 and p53. Promoter activity was normalized with respect to transfection efficiency as in Figure 1 and is shown as fold activation over the reporter alone. The results are the average and standard deviation of four experiments.

Over-expression of p53 has been shown to repress E2-activated transcription [27]. In order to determine whether p53 can repress transcription activation by an E2 protein defective in the induction of apoptosis in HPV-negative cells, we performed transcription assays using an E2 responsive reporter gene. Plasmids expressing wild type E2 or E2m1 were transiently transfected into HeLa cells along with the E2 responsive reporter described above. The results of this experiment are shown in Figure 3A. As can be seen from the data, the wild type E2 protein activates transcription (Fig. 3A, column 2). Co-expression of p53 with wild type E2 results in a reduction in E2-activated transcripion (Fig. 3A, column 3). Since the results of these experiments are normalised with respect to transfection efficiency using a co-transfected plasmid expressing the Renilla luciferase gene, this decrease in reporter activity cannot be due to increased cell death in the presence of E2 and p53. As expected, Em1 activates transcription to almost exactly the same level as wild type E2 (Fig. 3A, column 4). However, in this case co-expression of p53 and E2m1 has no effect on E2m1-activated transcription (Fig. 3A, column 5). These data suggest that the E2-p53 interaction is required for the down-regulation of E2-activated transcription. To determine whether this down-regulation is solely dependent upon p53 or whether down-regulation is mediated by p53 acting on E6 or E7, we repeated this experiment in SAOS-2 cells. As can be seen from the data shown in Figure 3B, p53 also down-regulates E2-activated transcription in these HPV-negative, p53-null cells. Furthermore, as seen in HeLa cells, p53 has no effect on E2-m1-activated transcription in these cells. These data confirm that p53 down-regulates E2-activated transcription in the absence of E6 and E7 and via interaction with E2.

### The E2-p53 interaction is not required for HPV DNA replication

**Figure 4:**
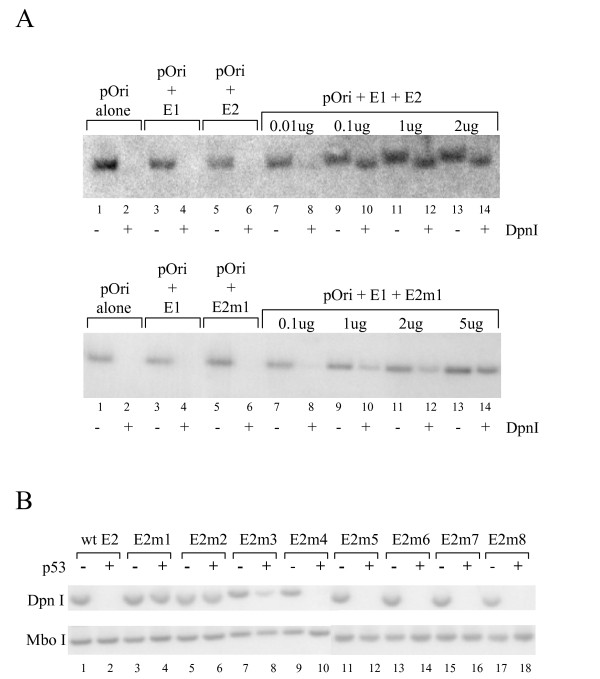
**p53 inhibits DNA replication mediated by E2**. (A) A transient HPV replication assay performed in U2OS cells co-transfected with a plasmid containing the HPV 16 origin of replication (pOri) and plasmids expressing HPV 16 E1 and the HPV 16 E2 protein (top panel) or HPV 16 E1 and the HPV 16 E2m1 protein (bottom panel) in the combinations and amounts shown. After 72 hours DNA was extracted from the cells and digested with XmnI to linearise pOri and either DpnI to remove unreplicated (input) DNA or MboI to remove replicated DNA. Linearised pOri was then detected by Southern analysis using a specific probe. The resulting autoradiograph is representative of several experiments. (B) A transient replication assay was performed exactly as described above but it this case pOri was co-transfected into U2OS cells with plasmids expressing wild type or mutated E2 proteins in the absence or presence of a plasmid expressing p53. The data shown is representative of several experiments.

To investigate whether the interaction between p53 and the C-terminal domain of E2 is required for HPV DNA replication, we performed transient replication assays in cells expressing wild type p53. A plasmid containing the HPV 16 origin of replication was transiently co-transfected into U2OS cells along with a plasmid expressing E2m1. After 72 hours, DNA was extracted from the transfected cells and digested with XmnI to linearise the plasmid containing the origin. The extracted DNA was then treated with DpnI in order to digest the unreplicated DNA or MboI in order to remove the replicated DNA and the digestion products analysed by Southern hybridisation. As expected, neither the origin containing plasmid alone, nor the origin containing plasmid co-transfected with plasmids expressing either E1 alone or E2 alone, replicate in this assay (Fig. 4A, top panel, lanes 1–6). However, in the presence of plasmids expressing E1 and E2, DpnI resistant and therefore replicated DNA is clearly detectable (Fig. 4A, top panel, lanes 7–14). Similarly, neither E1 alone nor E2m1 alone can bring about DNA replication in this assay (Fig. 4A, bottom panel, lanes 1–6). However, expression of E1 and E2m1 results in the production of replicated DNA (Fig. 4A, bottom panel, lanes 7–14). These data demonstrate that E2m1 is capable of facilitating HPV DNA replication in this assay.

### Repression of HPV DNA replication by p53

To examine the effect of p53 on replication mediated by the HPV 16 E1 and the E2 mutants, transient replication assays were performed with and without over-expression of p53. As expected given the results described in the previous section, in the absence of exogenous p53, all of the mutated E2 proteins are able to facilitate replication of the plasmid containing the viral origin (Fig. 4B). However, in the presence of over-expressed p53, replication mediated by the wild type E2 protein is abolished (Fig. 4B, lane 2). These data show that as in the case of the HPV 11 E2 protein, p53 is able to inhibit replication mediated by the wild type HPV 16 E2 protein. In marked contrast, over-expression of p53 does not inhibit replication mediated by the E2m1 or E2m2 proteins (Fig. 4B, lanes 4 and 6, respectively). Replication mediated by E2m3 is partially inhibited by over-expression of p53 (Fig. 4B, lane 8) whilst replication mediated by the remaining E2 mutants is completely abolished (Fig. 4B, lanes 9–18). These data suggest that the inhibition of replication by p53 is dependent on the interaction between p53 and the C-terminal domain of E2. However, since p53 and E2 have been shown to induce apoptosis in several cell lines, another explanation for these results could be that the cells expressing both proteins are dying or dead. In order to rule out this possibility we performed apoptosis assays using coverslips taken from same dishes of cells used for the replication assays. However, we did not see any increase in the number of U2OS apoptotic cells in the transfected population (data not shown). This is perhaps not unexpected since U2OS cells show defects in p53-induced apoptosis [35].

## Discussion

Although p53 has been found in replication centres associated with SV40, HSV, CMV and adenovirus, the role that p53 plays in the replication of these viruses is not well understood [36-39]. However, p53 has been shown to enhance the fidelity of DNA synthesis by HIV and murine leukaemia virus reverse transcriptase [40,41]. These observations suggest that as is the case in cellular replication, p53 may be involved in DNA replication and/or repair processes that are central to viral replication. The HPV 16 and 18 E6 proteins induce the degradation of cellular p53 and thereby reduce p53 levels. In contrast, the alternatively spliced E6 variant E6* inhibits E6-mediated degradation of p53 [42]. Furthermore, the low levels of p53 found in HPV-transformed cells are sufficient to allow these cells to undergo p53-dependent apoptosis [43]. This implies that p53 is functional in HPV-infected cells albeit at reduced levels.

It has been shown previously that p53 can inhibit HPV 11 replication [28,29]. Here we have shown that p53 can also inhibit HPV 16 DNA replication. The HPV 16 E2 protein can induce apoptosis in HPV-transformed cells and in some non-HPV-transformed cell lines [24]. In HPV transformed cells, E2-induced apoptosis (and E2-induced cell senescence) occurs via the reimposition of transcriptional control over the HPV E6 and E7 oncogenes [23,44,45]. However, in non-HPV transformed cells, HPV 16 E2-induced apoptosis does not require the ability of E2 to regulate transcription but instead requires the ability of E2 to interact with p53 [26]. Here we have shown that two mutant HPV 16 E2 proteins that fail to induce apoptosis in when co-expressed with p53 in SAOS-2 cells (E2m1 and E2m2), are still capable of inducing apoptosis in HPV-transformed cells. Although p53 can repress HPV DNA replication mediated by the wild-type E2 protein, p53 has no effect on HPV DNA replication mediated by these mutated E2 proteins. Similarly, although p53 can repress transcription activation by the wild-type E2 protein, p53 has no effect on transcription activation by E2m1. These data suggest that p53 represses HPV 16 DNA replication via interaction with the HPV 16 E2 protein. However, although the HPV 18 E2 protein binds to p53 in cells and the C-terminal domains of the HPV 18 and 16 E2 proteins bind to p53 *in vitro*, the C-terminal domain of the HPV 11 E2 does not bind to p53 [26]. Furthermore, the HPV 11 E2 proteins do not induce apoptosis in HPV-transformed cells or in non-HPV-transformed cells [26,46]. This suggests that p53 might inhibit HPV 11 DNA replication by another mechanism.

The series of E2 mutants created in this study are all able to induce apoptosis and activate transcription in HPV-transformed HeLa cells. Similarly they are all able to activate transcription in non-HPV-transformed SAOS-2 cells. However, they show interesting differences in their ability to induce apoptosis in non-HPV-transformed cells. The E2m1 mutant has residues D338, W341 and D344 mutated to alanine (Fig. 1B) and has been shown to be deficient in binding to p53 [26]. The E2m2 mutant has the three mutated residues in E2m1 with the addition of the mutation of E340 to alanine. Both of these mutants fail to induce apoptosis in SAOS-2 cells. However, E2m3 has residues E340, W341 and D344 mutated to alanine and this protein is able to induce apoptosis in SAOS-2 cells. This suggests that D338 plays an important role in the interaction. E2m5, E2m6, E2m76 and E2m8 each have single point mutations at D338, E340, W341 and D344 respectively, and are all able to induce apoptosis in SAOS-2 cells. This suggests that none of these single amino acid changes is sufficient to block the interaction with p53. Presumably p53 binds to E2 over a relatively large surface area that can tolerate these mutations.

The down regulation of HPV replication by p53 might be a cellular mechanism that acts to limit viral infection. However, it is more likely that the recruitment of p53 might be of some benefit to the virus. One possibility is that p53 might enhance the fidelity of HPV DNA replication or facilitate the repair of damaged viral DNA. However, we have been unable to detect any effect of p53 on the fidelity of HPV DNA replication (CB and KLG, unpublished observations). It is possible that the recruitment of p53 might have a subtle effect on the viability of this virus. A detailed study of the viral life cycle will be required in order to reveal any such effect.

## Conclusion

Our data suggest that p53 can down-regulate HPV 16 DNA replication via an interaction with the viral E2 protein. Disruption of the E2-p53 interaction alleviates the negative effect of p53 on HPV DNA replication. Further studies will be required to determine the role this interaction plays in the HPV life cycle.

## Methods

### Plasmids used in this study

The HPV 16 E2 expression vector pWEB-E2 and the vector expressing E2m1 (pWEB-E2p53m) have been described previously [26]. The mutated region of E2 is encoded between unique PstI and AflII restriction sites in pWEB-E2p53m. Using mutagenic PCR primers that flank these restriction sites further mutated DNA fragments were produced. After digestion with Pst1 and AflII the mutated sequences were inserted into pWEB-E2p53m cut with the same enzymes. All constructs were sequenced in order to confirm that the required mutations had been introduced. Plasmid pCMV-E1_16 _expresses the HPV 16 E1 protein [47]. pCB6-p53 expresses the full length p53 protein and was a kind gift from Dr Anne Williams (University of Bristol). The E2-responsive reporter plasmid pGL3-tk6E2 contains six E2 binding sites upstream of the minimal thymidine kinase promoter and firefly luciferase gene [32]. pRL-CMV (Promega) expresses Renilla luciferase under the control of the CMV enhancer and early promoter. pEGFP-C1 expresses the green fluorescent protein. The plasmid p16Ori contains the origin of DNA replication from HPV 16 (nucleotides 7838 to 130) in a backbone of pKS(-) BluescriptII (Stratagene) [47].

### Cell lines and transient transfection

All cell lines were maintained in Dulbecco's modified Eagle's medium supplemented with 10% FBS and penicillin (10^5 ^units/L) and streptomycin (100mg/L), and maintained at 37°C in 5% CO_2_. For apoptosis and transcription assays cells were transfected using Fugene 6 (Roche) at a ratio of 2:1 Fugene 6 (ml): DNA (mg) as directed by the manufacturer. For transient DNA replication assays cells were transfected using calcium phosphate precipitation.

### Apoptosis assays

Twenty-four hours prior to transfection 3.5 × 10^5 ^cells were seeded onto coverslips in six-well plates. The cells were transiently co-transfected with E2 and p53 expression vectors along with a GFP expression vector used in order to identify transfected cells. Thirty hours post-transfection the cells were fixed with 4% paraformaldehyde in PBS at 22°C for 30 minutes. Following further washes with PBS, the cells were stained with bisbenzimide (Hoechst No.33258: Sigma) for 30 minutes before being washed in PBS and mounted onto microscope slides in MOWIOL (Calbiochem). Fluorescence microscopy was carried out using a Leica DM IRBE inverted epi-fluorescent microscope fitted with FITC and DAPI filter sets and a 20× air objective (Leica). Apoptotic cells were identified on the basis of their morphological characteristics: membrane blebbing, chromatin condensation and the formation of apoptotic bodies. At least 100 GFP-expressing cells were counted per coverslip and the number of apoptotic cells within this population recorded and the percentage of apoptotic cells calculated.

### Transcription assays

Twenty-four hours prior to transfection 7 × 10^5 ^cells were seeded onto 60 mm diameter dishes. The cells were then transiently transfected with expression and reporter plasmids using Fugene 6. Twenty-four hours post-transfection the cells were washed three times with PBS and lysed in passive lysis buffer (Promega) for 20 minutes at 22°C. The dishes were then scraped and the lysates collected. Following centrifugation for 1 minute at 12,000 rpm in a microcentrifuge to pellet debris, 20 μl of lysate was removed and assayed for luciferase activity using a Berthold Technologies luminometer and dual luciferase assay system (Promega).

### Transient replication assays

Twenty-four hours prior to transfection 3 × 10^5 ^cells were seeded in 100 mm diameter dishes. The cells were then transiently co-transfected with the HPV 16 origin containing plasmid pOri, E1- and E2-expression vectors and in some cases, a p53 expression vector by calcium phosphate precipitation. Seventy-two hours post-transfection, the cells were washed twice in PBS and low molecular weight DNA extracted using the Hirt method [48]. The extracted DNA was linearised by digestion with XmnI. 90% of the linearised DNA was then digested with DpnI to remove the input DNA. The remaining 10% of the linearised DNA was digested with MboI to remove replicated DNA, leaving linearised input DNA (input DNA is resistant to MboI digestion). The MboI digested and DpnI digested samples were electrophoresed on a 0.8% agarose gel and analysed by Southern blot using an HPV 16-specific probe. Hybridising bands were detected using a Molecular Dynamics PhosphorImager and ImageQuant 3.3 software.

## Competing interests

The author(s) declare that they have no competing interests.

## Authors' contributions

CB carried out the mutagenesis and apoptosis assays. CB and AMK carried out the transcription assays. CB, ERT and IMM performed the replication assays. KG wrote the manuscript.
